# Culture-independent analysis of bacterial diversity in a child-care facility

**DOI:** 10.1186/1471-2180-7-27

**Published:** 2007-04-05

**Authors:** Lesley Lee, Sara Tin, Scott T Kelley

**Affiliations:** 1 Department of Biology, San Diego State University, San Diego, California, USA

## Abstract

**Background:**

Child-care facilities appear to provide daily opportunities for exposure and transmission of bacteria and viruses. However, almost nothing is known about the diversity of microbial contamination in daycare facilities or its public health implications. Recent culture-independent molecular studies of bacterial diversity in indoor environments have revealed an astonishing diversity of microorganisms, including opportunistic pathogens and many uncultured bacteria. In this study, we used culture and culture-independent methods to determine the viability and diversity of bacteria in a child-care center over a six-month period.

**Results:**

We sampled surface contamination on toys and furniture using sterile cotton swabs in four daycare classrooms. Bacteria were isolated on nutrient and blood agar plates, and 16S rRNA gene sequences were obtained from unique (one of a kind) colony morphologies for species identification. We also extracted DNA directly from nine representative swab samples taken over the course of the study from both toy and furniture surfaces, and used "universal" 16S rRNA gene bacterial primers to create PCR-based clone libraries. The rRNA gene clones were sequenced, and the sequences were compared with related sequences in GenBank and subjected to phylogenetic analyses to determine their evolutionary relationships. Culturing methods identified viable bacteria on all toys and furniture surfaces sampled in the study. *Bacillus *spp. were the most commonly cultured bacteria, followed by *Staphylococcus *spp., and *Microbacterium *spp. Culture-independent methods based on 16S rRNA gene sequencing, on the other hand, revealed an entirely new dimension of microbial diversity, including an estimated 190 bacterial species from 15 bacterial divisions. Sequence comparisons and phylogenetic analyses determined that the clone libraries were dominated by a diverse set of sequences related to *Pseudomonas *spp., as well as uncultured bacteria originally identified on human vaginal epithelium. Other sequences were related to uncultured bacteria from wastewater sludge, and many human-associated bacteria including a number of pathogens and opportunistic pathogens. Our results suggest that the child-care facility provided an excellent habitat for slime-producing Pseudomonads, and that diaper changing contributed significantly to the bacterial contamination.

**Conclusion:**

The combination of culture and culture-independent methods provided powerful means for determining both viability and diversity of bacteria in child-care facilities. Our results provided insight into the source of contamination and suggested ways in which sanitation might be improved. Although our study identified a remarkable array of microbial diversity present in a single daycare, it also revealed just how little we comprehend the true extent of microbial diversity in daycare centers or other indoor environments.

## Background

Child-care facilities appear to provide a setting with many opportunities for exposure and transmission of bacteria and viruses [1-4]. Preschool aged children are often sick with illnesses of unknown origins, and young children have not yet mastered the sanitary cleaning habits present among most adults in our society. Moreover, children have had less exposure to microorganisms, making them more likely to catch and transmit pathogens or opportunistic pathogens, and perhaps more likely to suffer ill effects from contact in densely populated facilities. Recent studies of microbial diversity in indoor environments using molecular methods have revealed considerable bacterial contamination and underscored how little we know about such contamination [5-7]. Understanding the potential public health risks in daycare centers requires a better understanding of microbial diversity in these settings. This is particularly important given the increasing reliance of working parents on daycare facilities for childcare [4].

Culture-based studies of human indoor environments have shown that significant levels of bacteria are present in seemingly innocuous areas such as office buildings, residential homes, and children's schools and daycare centers [8-10]. According to these surveys, low DNA G+C content, Gram-positive bacteria, such as *Bacillus cereus*, *Bacillus licheniformis*, *Brevibacillus brevis *and *Staphyloccus *spp. along with a few Gram negative species including *Chryseomonas *spp. and *Pantoea *spp. tend to predominate [8,11,12]. Indoor culturing studies have also identified the presence of bacteria from the order Actinomycetes, including *Rhodococcus fasclans*, *Arthrobacter pascens*, and *Corynebacterium *spp. [8,11].

Recently, culture-independent molecular studies have greatly expanded our understanding of the bacterial diversity that can be present in indoor environments. The molecular methods we performed in this study included PCR amplification of 16S rRNA genes conducted on DNA extracted directly from our environmental samples. Culture-independent methods have been able to offer a much more complete view of the bacteria present in ordinary everyday surroundings such as indoor pools, shower curtains, and airplane bathrooms; these same methods should prove equally effective for use in daycare settings [5-7]. In some cases, culture-independent methods have identified the source of illness when the microbes were unknown or not currently culturable [5,13].

In an environment so potentially rich in microbial diversity, culturing methods readily identify bacteria with known growth requirements and these methods are necessary to prove the viability of microorganisms in the environment. However, previous work has shown that <1% of bacterial species in a given environment are culturable suggesting that a vast majority of the true diversity may be missed if studies rely solely on culturing [14,15]. Indeed, the development of culture-independent methods based on the 16S rRNA gene used in conjunction with phylogenetic analysis has revealed an abundant array of previously unknown and uncultured microbes, including entirely new bacterial divisions [13-18]. The 16S rRNA gene is particularly useful for molecular analysis and identification of organisms due to its high level of information content, conserved nature, and it's presence in all cellular microorganisms [15]. Researchers have also begun to use culture-independent methods to study human biology [19-22], complex diseases [13,23], and human environments [5-7]. Collectively these studies have exposed a remarkable array of microorganisms, many of them with no cultured representatives. In the case of human environments, many potentially opportunistic pathogens have been identified [5-7].

In this study, we surveyed the bacterial diversity present in a daycare facility using both culture and culture-independent methods to analyze samples taken from various toys and surfaces (e.g., counter-tops). This allowed us to gauge the overall complexity of bacterial diversity, determine viability of bacteria, and see how the diversity and abundance changed over time. A total of four rooms were sampled over a six-month period. Sampling was alternated between two toddler rooms and two infant rooms every one to two weeks. Of these samples, DNA was successfully extracted directly from nine swabs, and the samples were subjected to both culture and culture-independent analysis. The facility tested in this case had specific disinfection protocols in place for daily cleaning of the rooms and washing of the toys that the children have played with or come into contact with during the course of the day. Cleaning protocols (e.g., cleaning surfaces with 10% bleach) were followed diligently by the staff in this daycare facility, which placed a high premium on cleanliness.

## Results

Table 1 details the total number of cultured isolates obtained over the course of the study based on the16S rRNA gene sequence analysis and visible colony morphology. The lysozyme extraction protocol effectively isolated bacterial DNA from all the colonies picked from plates. Typical DNA yields for bacterial colonies were in the range of 0.6 to 82.4 ng μl^-1^. Table 2 shows the results of culturing on the environmental swab samples taken between September 2005 – April 2006. For all days sampled except October 6, 2005, colonies grew on either blood or nutrient agar plates. This means that there were large numbers of viable bacteria consistently present on the surfaces and toys sampled at the daycare center. We isolated as many as 29 putative bacterial species. We considered it a potentially different species of bacteria if it had a unique 16S rRNA gene sequence or a clearly distinct morphology or both (Table 2). *Bacillus *species were the most commonly culture-isolated bacteria, followed by *Staphylococcus *spp. Culture methods identified at least 29 viable bacterial species on toy and furniture surfaces over the 6 months of the study (Table 1). Species of *Bacillus *were isolated every day of sampling (Table 2), and we identified as many as 15 different distinct morphologies over the course of the study (Table 1).

**Table 1:** Unique morphologies present in culture results, and their identification through sequencing

Organism	**Sequence Length**^1^	**% Identity**^2^	GenBank Accesssion	Morphology
*Acinetobacter *sp.	741	98	EF409307	Acinet. 1
*Bacillus licheniformis*	720	99	EF409308	Bacillus M1
*Bacillus megaterium*	739	99	EF409309	Bacillus M2
*Bacillus *sp.	498	97	EF409310	Bacillus M3
*Bacillus *sp.	754	100	EF409311	Bacillus M4
*Bacillus *sp.	724	99	EF409312	Bacillus M 5 (Nutrient)
*Bacillus *sp.	748	99	EF409313	Bacillus M6
*Bacillus *sp.	730	100	EF409314	Bacillus M7
*Bacillus *sp.	664	99	EF409315	Bacillus M8
*Bacillus *sp.	741	89	EF409316	Bacillus M9
*Bacillus *sp.	677	98	EF409317	Bacillus M10
*Bacillus *sp.	721	99	EF409318	Bacillus M11
*Bacillus subtilis*	759	99	EF409319	Bacillus M12
*Bacillus subtilis*	759	100	EF409320	Bacillus M5a
*Bacillus subtilis*	736	99	EF409321	Bacillus M13
*Bacillus subtilis*	722	99	EF409322	Bacillus M14
*Bacillus subtilis*	718	99	EF409323	Bacillus M15
*Brevibacillus *sp.	711	99	EF409324	Brevi. M1a
*Brevibacterium *sp.	695	99	EF409325	Brevi. M1b
*Enterococcus faecalis*	764	99	EF409326	Enterococ. M1
*Exiguobacterium *sp.	767	99	EF409327	Exiguobac. M1
*Microbacterium aurum*	289	99	EF409328	Microbac. M1a
*Microbacterium esteraromaticum*	327	98	EF409329	Microbac. M1b
*Moraxella osloensis*	750	98	EF409330	Morax. M1
*Pseudomonas stutzeri*	682	99	EF409331	Pseudom. M1
*Staphylococcus aureus*	752	99	EF409332	Staph. M1
*Staphylococcus aureus*	727	100	EF409333	Staph. M2
*Staphylococcus epidermidis*	721	100	EF409334	Staph. M3
*Staphylococcus haemolyticus*	703	99	EF409335	Staph. M4

^1 ^Length of sequence used in BLAST search

^2 ^Based on BLAST alignment

^3 ^Abbreviations for each morphology type; cross-referenced in Table 2

**Table 2:** Culture results for environmental swab samples

Date	Plate type	Total Plates	**% Growth**^1^	**Organisms detected**^2^
30-Sep-2005	Nutrient	6	66.7	N/A
	Blood	4	75.0	N/A
6-Oct-2005	Nutrient	12	0.0	N/A
	Blood	12	33.3	N/A
14-Oct-2005	Nutrient	14	16.7	N/A
	Blood	0	0.0	N/A
21-Oct-2005	Nutrient	24	79.2	*Bacillus *sp (Bacillus M7)
	Blood	0	0.0	*Bacillus *sp. (Bacillus M2, M11)
28-Oct-2005	Nutrient	10	80.0	N/A
	Blood	6	83.3	N/A
3-Nov-2005	Nutrient	13	84.6	*Bacillus *sp. (Bacillus M7)
	Blood	12	83.3	*Bacillus *sp. (Bacillus M11)
15-Nov-2005	Nutrient	22	81.8	*Bacillus *sp. (Bacillus M7)
	Blood	16	56.3	*Bacillus *sp. (Bacillus M3, M11, 14)
18-Jan-2006	Nutrient	16	56.3	N/A
	Blood	0	0.0	N/A
25-Jan-2006	Nutrient	16	68.8	N/A
	Blood	16	75.0	N/A
3-Feb-2006	Nutrient	12	75.0	*Bacillus *sp. (Bacillus M2, M7, M10), *Staphylococcus *sp. (Staph. M1)
	Blood	16	81.3	*Bacillus *sp. (Bacillus M1, M2, M3, M14), *Staphylococcus *sp. (Staph. M2), *Moraxella *sp. (Morax. M1), *Brevibacterium *sp. (Brevi. M1b), *Brevibacillus *sp. (Brevi. M1a)
15-Feb-2006	Nutrient	10	90.0	*Bacillus *sp. (Bacillus M10), *Staphylococcus *sp. (Staph. M3)
	Blood	10	80.0	*Bacillus *sp. (Bacillus M1), *Microbacterium *sp. (Microbac. M1)
1-Mar-2006	Nutrient	10	70.0	*Pseudomonas *sp. (Pseudo. M1), *Bacillus *sp. (Bacillus M1, M5, M7), *Staphylococcus *sp. (Staph M3)
	Blood	10	60.0	*Bacillus *sp. (Bacillus M1, M5, M9, M11), *Staphylococcus *sp. (Staph. M2)
8-Mar-2006	Nutrient	20	85.0	*Bacillus *sp. (Bacillus M5, M7), *Staphylococcus *sp. (Staph. M3), *Enterococcus *sp. (Enterococ. M1)
	Blood	20	90.0	*Bacillus *sp. (Bacillus M1, M11), *Staphylococcus *sp. (Staph. M2), *Moraxella *sp. (Morax. M1)
29-Mar-2006	Nutrient	16	50.0	*Bacillus *sp. (Bacillus M5, M7), *Staphylococcus *sp. (M1), *Enterococcus *sp. (Enterococ. M1)
	Blood	16	50.0	*Bacillus *sp. (Bacillus M2, M5a, M11, M13), *Staphylococcus *sp. (M2)
5-Apr-2006	Nutrient	12	41.7	*Bacillus *sp. (Bacillus M7, M12), *Staphylococcus *sp. (Staph. M1, M3), *Acinetobacter *sp. (Acinet. M1)
	Blood	12	58.3	*Bacillus *sp. (Bacillus M2, M8, M11, M14), *Staphylococcus *sp. (M2, M3)
12-Apr-2006	Nutrient	16	37.5	*Bacillus *sp. (Bacillus M7, M12), *Brevibacterium *sp. (Brevi. M1b), *Pseudomonas *sp. (Pseudo. M1), *Exiguobacterium *sp. (Exiguobac. M1)
	Blood	16	43.8	*Bacillus *sp. (Bacillus M1, M2, M8, M11), *Staphylococcus *sp. (Staph. M2), *Brevibacterium *sp. (Brevi. M1a), *Exiguobacterium *sp. (Exiguobac. M1)
19-Apr-2006	Nutrient	16	43.8	*Bacillus *sp. (Bacillus M10, M12), *Acinetobacter *sp. (Acinet. M1), *Enterococcus *sp. (Enterococ. M1)
	Blood	16	81.3	*Bacillus *sp. (Bacillus M1, 2, M15), *Brevibacterium *sp. (Brevi. M1b), *Microbacterium *sp. (Microbac. M1a)

^1 ^Percentage of plates with microbial growth

^2 ^Observed morphologies (see Table 1); N/A = Not Available for that sampling day.

The lysozyme DNA extraction protocol proved effective for direct swab extractions and yielded DNA in the range of 5.1 to 14.7 ng μl^-1^. Bead-beating methods are typically preferred for isolating environmental DNA because the mechanical shearing of cells by the beads helps extract DNA from particularly "tough" bacterial cells such as bacterial vegetative cells (e.g., *Pseudomonas putida*), bacterial endospores (e.g., *Bacillus *spp.), and fungal conidia (e.g., *Fusarium moniliforme*) [24]. However, our attempts with bead-beating methods failed to isolate sufficient DNA from the swabs (data not shown), whereas the lysozyme method yielded sufficient amounts of DNA for PCR.

**Figure 1:**
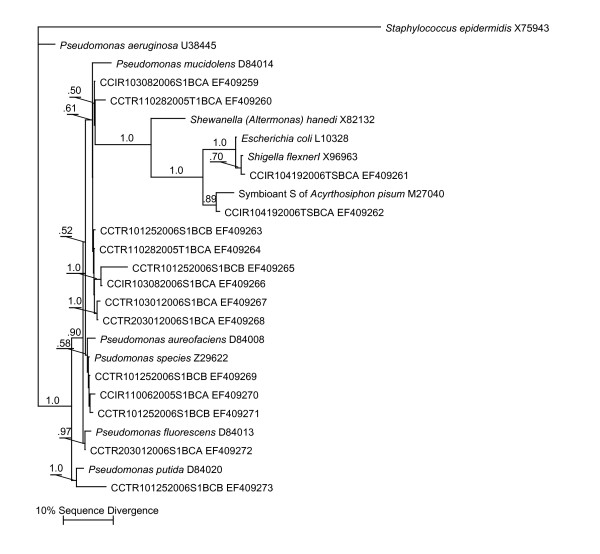
**Results of Bayesian phylogenetic analysis of *Pseudomonas*-related 16S rRNA gene sequences representing the most common clones found in PCR-amplified libraries from swabs samples of toys and surfaces**. Cloned sequences are indicated by "CCTR" (Children's Center Toddler Room) or "CCIR" (Children's Center Infant Room) prefixes followed by the date of sampling and whether the sequence was obtained from a toy (T) or a surface (S). The phylogeny includes sequences of closely related cultured and uncultured organisms. GenBank accession numbers are presented next to the names. The values above the branches indicate the Bayesian posterior probabilities (above 0.5) under the specified model of evolution for each node (see Methods for details). Maximum Parsimony and Maximum Likelihood analyses produced highly similar tree topologies. MP bootstrap values were similarly high at nodes well-supported by Bayesian analysis.

**Figure 2:**
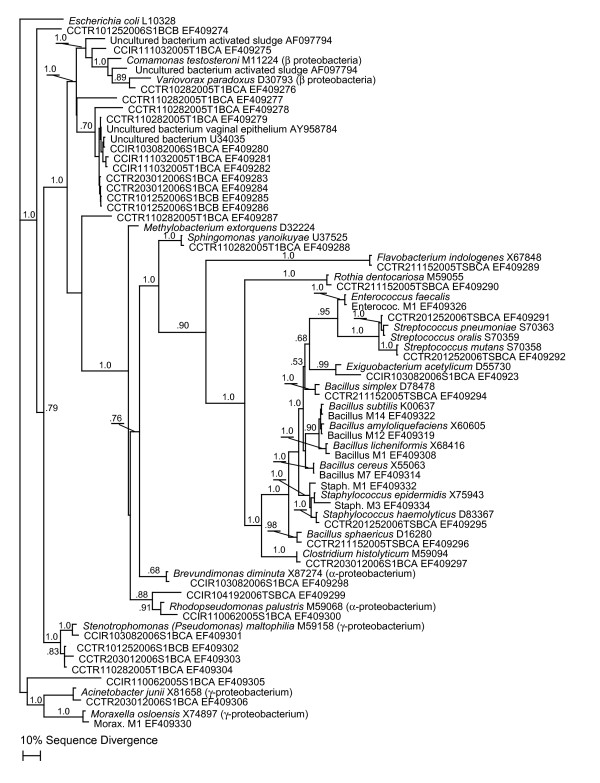
**Results of Bayesian phylogenetic analysis of 16s rRNA gene sequences from other phylogenetic groups of bacteria found in PCR-amplified clone libraries from swabs samples of toys and surfaces**. Cloned sequences are indicated by "CCTR" (Children's Center Toddler Room) or "CCIR" (Children's Center Infant Room) prefixes followed by the date of sampling and whether the sequence was obtained from a toy (T) or a surface (S). GenBank accession numbers are presented next to the names. The tree also includes sequences from some of the cultured isolates in Table 1. The values above the branches indicate the Bayesian posterior probabilities (above 0.5) under the specified model of evolution for each node. Maximum Parsimony and Maximum Likelihood analyses produced highly similar tree topologies. MP bootstrap values were similarly high at nodes well-supported by Bayesian analysis.

**Figure 3:**
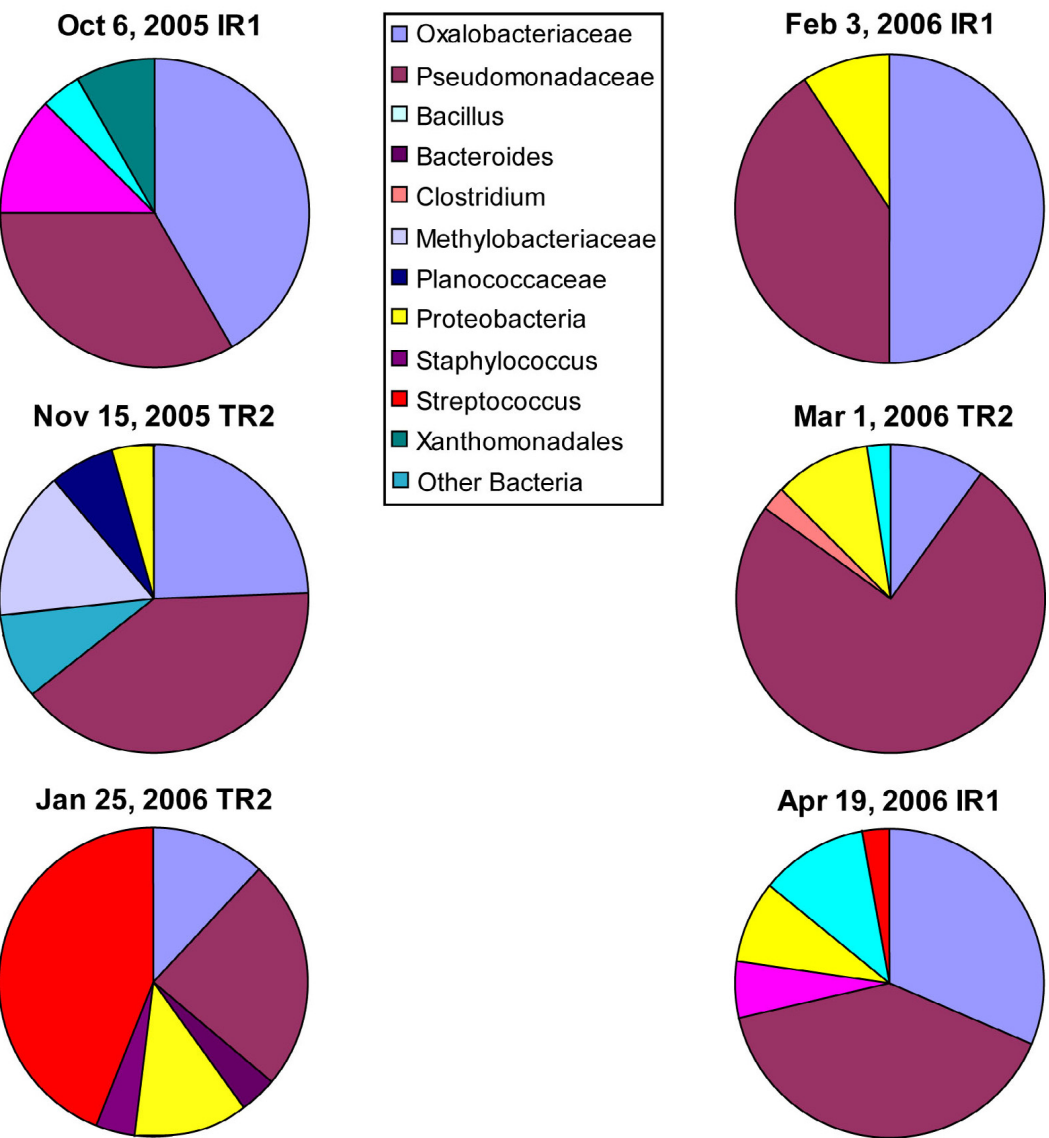
**Graphical representation of common bacterial-type abundance found in the six clone libraries made from furniture surface swabs based on 16S rRNA gene sequence identification**. Pseudomonads and uncultured *Oxalobacteraceae *were consistently found on all surfaces at high levels. *Bacillus *species were also common, though at lower abundance. One date in particular, Jan 25^th^,(Toddler Room 2) had a particularly high concentration of *Streptococcus*-related sequences.

Figure 1 and 2 show the results of phylogenetic analysis of selected sequences from the 453 clones obtained from the nine libraries relative to sequences from cultured and uncultured bacteria from other studies. Based on our survey of nine 16S rRNA gene PCR-clone libraries we identified as many as 190 bacterial species (1% divergence; 141 at the more conservative 3% divergence level), most of them with no cultured representatives. The clone library sequence coverage ranged from 48% to 65% (average 54%) for the nine clone libraries. Most sequences found in both groups appeared to be uncultured bacterial species. Members of the *Pseudomonadaceae *and the *Oxalobacteraceae *predominated in the clone libraries (Fig. 3). Pseudomonads were particularly abundant and were on every surface sampled on every sampling occasion (Fig. 3).

Since the main purpose of this study was to identify the types of organisms in the daycare center, one-directional sequencing of the first part of the small-subunit rRNA gene, which includes the most variable part of the gene, was enough for our purposes. Sequencing bi-directionally would have been ideal in terms of reducing error, but would have doubled the sequencing costs and added little to our understanding of the diversity. Also, we trimmed the sequences to around ~500 bp in length, and edited out the most problematic part of the reads. Any errors that remained would have had little impact on the phylogenetic analysis. However, we have made glycerol stocks of all the clone libraries we created, which are available from the authors upon request.

The alignment of the edited and trimmed sequences proved straightforward, and the alignment was checked by confirming complementary base-pairing in known stem regions of the alignment. A total of 78 sequences were deposited in GenBank, including sequences from both culture isolates (Table 1) and representative clone library sequences used in the phylogenetic analysis (Fig. 1, 2). Approximately 500 nucleotide positions, corresponding to *E. coli *positions 25 to 534, were used for all the phylogenetic analyses. There was strong support for the majority of the phylogenetic relationships as judged by both Bayesian posterior probabilities (Fig. 1, 2) and MP bootstrap support (not shown). Bayesian, MP and ML methods produced highly similar tree topologies. The differences in tree topologies produced by the various methods were in regions of the trees not supported by either posterior probabilities (< 0.5) or MP bootstrap values (<50%). The phylogenetic analysis allowed us to easily identify the position of our cloned environmental sequences within known bacterial divisions (Fig. 1, 2). Using this information, and the Fastgroup II dereplication information, we were able to assess the relative abundance of various sequences in clone libraries and these are presented in Figure 3.

## Discussion

Our combination of culturing and culture-independent techniques revealed a remarkable diversity of bacteria contaminating every surface sampled in the daycare facility. *Bacillus *spp. were particularly common (Table 1 and 2). *Bacillus *endospores disperse rapidly through the air and are ubiquitous in soils and other environments [25,26] so their abundant presence here was not alarming. *Staphylocccus *species were also repeatedly isolated, as were species of *Pseudomonas *and *Microbacterium *(Table 1 and 2). A number of the isolates were potential pathogens and opportunistic pathogens, including *Enterococcus faecalis *[27]*, Moraxella osloensis *[28], and *Staphylococcus haemolyticus *[29]*. E. faecalis *has become a particular problem in hospitals [27]. Species belonging to these genera of bacteria are commonly found on skin, nostrils, or even as part of the normal gut microbiota [30]. Normal shedding of these surfaces, along with the attached bacteria, may explain their abundance in indoor human environments [9,30].

Our culturing results appeared highly similar to culture-based studies of other indoor environments [8,9]. Specifically we found ~90% of the same bacterial genera as another culture-based study of a daycare setting [8]. Most of these studies sampled airborne bacteria in environments, such as daycare centers, schools, and office buildings [8,9,11,12]. Our results suggest diversity found in air-sampling studies is very similar to that of surface sampling methods and may be a reasonable substitute for costly air-sampling methods at least in terms of determining microbial diversity.

Although the culture-based methods discovered a number of bacterial species and confirmed the viability of bacteria on surfaces in the daycare facility, these methods identified only a small fraction of the true bacterial diversity (~3%). The culture-independent portion of our analysis revealed a whole new dimension of largely unexplored microbial diversity present in a daycare center environment. Similar to other culture-independent studies in human environments [5-7], we uncovered an extraordinary diversity of bacteria from 16 bacterial divisions or sub-divisions that included many bacterial species without cultured representatives (Fig. 1, 2, 3).

The largest proportion of sequences found in the clone libraries came from two groups: the *Pseudomonadaceae *and the *Oxalobacteraceae *(Fig. 3). Pseudomonads comprise an extremely diverse array of bacteria that grow on numerous carbon sources and are often associated with spoilage [31]. Many of them produce biofilm "slime layers" that serve as environmental protection and make them resistant to both antibiotics [32] and cleaning regimens [33]. Moreover, this same slime production ability appears to protect them from the mammalian immune system [32]. A number of Pseudomonads, such as *P.stutzeri*, are known opportunistic pathogens [34] and have also been implicated in hospital acquired infections [35,36]. *P. aeruginosa *is also known to be resistant to antibiotics [37].

The predominance of a diverse array of Pseudomonads in the daycare appears to be quite consistent with the nature of the environment. The constant spillage of food and liquids, spread over every surface reachable by children, would make a perfect growth medium for Pseudomonads [31]. This particular daycare center had very rigorous cleaning policies. However, the natural resistance of Pseudomonads to cleaning may actually have served to increase their abundance relative to other bacteria. Given the abundance of Pseudomonads in our clone libraries, it is somewhat surprising that we did not find more species growing on nutrient agar plates (Table 2). Another published culture-based study of a daycare facility showed a lack of diversity on nutrient agar plates [8]. However, growth on media other than blood or nutrient agar was not tried. It is possible that had a media specifically designed to culture Pseudomonads been selected, our results may have been different. In addition, our inability to grow these bacteria on agar plates may be a result of the fact that so many of the *Pseudomonas *spp.-related sequences came from uncultured bacteria (1–3% divergence from cultured species; Fig. 2).

The other most consistently abundant group, based on clone library sequence analysis, included a large collection of uncultured species in the *Oxalobacteraceae *family. According to the research literature, the *Oxalobacteraceae *include numerous bacterial species with diverse habitats. For example, many *Collimonas *and *Herbaspirillum *spp. are soil dwelling bacteria [38], while *Oxalobacter formingenes *is found in the human gastrointestinal tract [39]. Unfortunately, our uncultured species appeared to belong to a novel group of *Oxalobacteraceae *leaving us with little information concerning their source or habitats, apart from a rather basic understanding of their phylogenetic relationships. However, a recent culture-independent study of human-associated microbial communities [40] allowed us to identify the human vaginal epithelium as a likely source of a large number of these sequences (Fig. 1, 3). An intensive sequencing effort by Hyman et al. (2005) revealed a tremendous diversity of uncultured bacteria associated with the vaginal epithelium, and many of the 16S rRNA gene sequences obtained from surface samples, especially within the *Oxalobacteraceae*, were closely related to these published sequences (Fig. 2). Sequences in our clone libraries from nine other bacterial divisions were also nearly identical to bacterial sequences isolated from the vaginal epithelium (Fig. 2). We also note that we found many sequences of apparently uncultured bacteria related to uncultured bacteria identified from molecular studies of wastewater sludge (Fig. 2; [41,42]).

The predominance of sequences in our libraries related to bacteria found in the vaginal epithelium and in wastewater sludge suggests that a significant proportion of the bacterial contamination in daycares results from frequent diaper changes. This conclusion is supported by the discovery of cultured bacteria known to reside in the human intestine (e.g., *E. coli*, *Enterococcus faecalis*; Fig. 2). Since we know so little about the uncultured bacteria, we cannot say whether they pose a particular health threat. However, the significant diversity of so many human-associated bacteria contaminating various toys and surfaces suggests that enteropathogenic bacteria could be easily spread in daycare settings. Given the fact that we did not achieve full sampling coverage of the sequence diversity for any of the samples (average clone library sequence coverage ~54%), we expect that many more bacterial species may be found in daycare settings. These results not only demonstrate how little is known about microbial diversity of indoor environments, but also emphasizes the need for a much more complete understanding of microbes associated with humans, which appeared to be the source of most of the contamination.

In addition to the sequences from uncultured bacteria, we also identified a number of sequences in the culture-independent molecular analysis with close relatives known to be pathogens or opportunistic pathogens that were not found through culturing. These include sequences related to *Streptococcus mutans*, *S. mitis*, *Chryseobacterium indologenes *[43], *Stenotrophomonas maltophilia *[44,45]*, Flavobacterium indologenes *[46] and *Rothia dentocariosa *[47] (Fig. 1, 2). The January 25 sampling date in particular had a particularly high abundance of *Streptococcus*-related species (Fig. 3). This sampling was right in the middle of the cold and flu season and was around the time a large number of the kids were kept home due to illness (Daycare staff, pers. comm.). The fact that we found so few of these species through standard culturing approaches supports that notion that culture-independent molecular analyses provide a powerful additional means for studying indoor environments and possibly identifying infectious agents.

The results of this study should also be examined from a public health viewpoint, in order to assess which organisms have the greatest potential to cause illness in children. *Streptococcus pneumoniae *and *Staphylococcus epidermidis *were identified in several samples, these represent known pathogens that can cause pneumonia and meningitis in children [48,49]. *Stenotrophomonas maltophilia *was also found, this bacteria has been known to cause infections and bacteremia in pediatric patients who have contracted it as a nonsocomial infection during a hospital stay [50,51]. The presence of *Stenotrophomonas maltophilia *was also somewhat distressing due to the recent emergence of an antibiotic resistant strain [52]. Other pathogenic strains potentially harmful to children that were found included *Rothia dentocariosa*, known to cause the childhood illness tonsillitis [53], *Enterococcus faecalis*, a bacteria associated with urinary tract infections [54,55], and *Shigella flexneri*i, which causes the common childhood ailment of acute diarrhea [56,57].

Although culture-independent methods have proven highly useful for uncovering a vast array of new microbes in many environments, including the daycare center, a number of authors have pointed out that methods based on amplifying 16S rRNA gene sequences using "universal" primers may not accurately reflect the true underlying diversity of a given environment [58,59]. Problems such as PCR-bias, ribosomal DNA copy number and the efficiency of DNA extraction procedures all have the potential to significantly skew abundance estimates and there may not be a direct relationship between the number of sequences of a particular type in a clone library and the number of organisms in the environment.

Nevertheless, as demonstrated in this paper, the culture-independent methods do allow for a much more comprehensive assessment of microbial diversity than culturing alone and provide an approximation of the relative diversity in the samples. Given the proper set of growing conditions, many of the uncultured bacteria could be isolated using culture-based methods. For example, by culturing for a longer period of time (five or more days), by culturing at a broader range of temperatures (e.g., 30°C), or by using alternative media (e.g., low-nutrient medium R2A) we might have isolated more types of bacteria. Longer incubation times would have been particularly helpful for growing organisms that tend to live in biofilms. Indeed, the culture-independent methods provide an excellent complement to the culturing approaches. Before the study, we did not expect to find such a diverse array of *Pseudomonadaceae *and *Oxalobacteraceae*, but with the knowledge gained from the culture-independent methods we can now adjust our culturing methods to grow these organisms.

## Conclusion

The diversity of bacteria in the daycare environment appears to be a rich combination of bacterial species associated with both humans and the outside environment (e.g., *Bacillus *in soils). Given the extremely high bacterial diversity, and the relatively low sequence coverage we achieved in this preliminary study (~54%), the overall diversity is almost certainly higher than we report. Our results suggest that the microbial diversity associated with human environments remains extremely poorly characterized. In terms of public health, we believe greater attention needs to be paid to the microbial contamination of environments (e.g., daycare centers, nursing homes and hospitals) that take care of the most vulnerable members of society. In terms of the child-care facilities per se, our results suggest that diaper changing stations should be moved further away from the play areas, and that more efforts should be focused on removing tough biofilms. Faster and more comprehensive culture-independent methods, such as environmental microarrays and metagenomic approaches, could help better understand the public health risks in these environments.

## Methods

### Sample Collection

Samples were collected from 4 different classrooms where children ages 0–4 years were taught daily. Environmental samples were taken with dual tip sterile cotton swabs (BBL CultureSwab™, catalog # 220135, Becton Dickinson, Sparks, MD) and these were stored in sterile-labeled tubes for immediate transport back to the lab. Toys and surfaces were sampled on a fixed surface area of approximately 13 cm^2^. During the course of the study the same furniture surfaces were sampled repeatedly, while the toys varied between samplings.

### Bacterial Culturing Methods

Immediately upon return to the lab, one tip of the dual-tip swab samples was placed into 7 mL of nutrient broth (Difco™, Becton Dickinson, Sparks, MD) and allowed to incubate overnight at 37°C. The second tip of each dual-tip swabs was labeled and placed at -80°C for later analysis. Overnight cultures of nutrient broth (Difco™) were used to streak 5% blood (Blood Agar Contact Plate, Hardy Diagnostics, Santa Maria, CA) and nutrient agar plates (Difco™, Becton Dickinson, Sparks, MD), which were also incubated overnight at 37°C. To minimize outside contamination, all culturing was performed in a biological hood using sterile instruments. The following day, plate growth and colony morphology were evaluated and recorded.

### DNA Extraction and PCR Amplification of Colonies and Swabs

DNA was extracted using a lysozyme-extraction protocol [60] directly from the bacterial colonies picked off plates using a sterile toothpick. One colony was selected from each observed morphology type. We used the same protocol to isolate DNA directly from the swabs (the environmental samples) for culture-independent analysis.

For the environmental extractions, cotton from the swab samples was removed using a sterile razor blade and placed into the lysozyme reaction mixture. The reaction mixture had a total volume of 200 μl and included the following final concentration: 20 M Tris, 2 mM EDTA (pH 8.0), 1.2% P40 detergent, 20 mg ml^-1 ^lysozyme, and 0.2 μm filtered sterile water (Sigma Chemical Co., St. Louis, MO). We used the same reaction buffer and extraction method for isolating DNA from the cultured organisms (Table 1). For the cultured bacteria, we used a sterile toothpick to pick a single colony from the agar plates, which was then swirled into the reaction mixture. Samples were incubated in a 37°C water bath for thirty minutes. Next, Proteinase K (DNeasy Tissue Kit, Qiagen Corporation, Valencia, CA) and AL Buffer (DNeasy Tissue Kit, Qiagen Corporation, Valencia, CA) were added to the tubes and gently mixed. Samples were incubated in a 70°C water bath for 10 min. All samples were subjected to purification using the DNeasy Tissue Kit.

Following extraction, the DNA was quantified using a NanoDrop ND-1000 Spectrophomtometer (NanoDrop Technologies, Willmington, DE). We created PCR-based clone libraries from nine of the swabs collected over the course of the study. With one exception, swabs we chose came from the same surface type (the toy shelf). Six of the swabs were selected to represent each month from October 2005 through April 2006, while the other three were selected as duplicates for three of the sampling days to determine the consistency of contamination across surfaces. This strategy allowed us to find the most consistently abundant types of bacteria and to detect any significant changes in diversity that might occur during the "cold and flu" season.

The PCR reactions utilized published bacterium specific primers primers 8F (5'-AGAGTTTGATCCTGGCTCAG-3') and 805R (5'-GACTACCAGGGTATCTAATCC-3') to amplify the 16S rRNA gene. The ~800 bp PCR products from amplification using these primers includes a portion of the 16S rRNA gene that has been shown to be particularly useful for database analysis and identification of bacterial sequences [13]. PCR was carried out in a total reaction volume of 50 μl including 1 μl (approx. 10 ng μl^-1^) of sample DNA as template, each deoxynucleoside triphosphate at 200 μM, 1.5 mM MgCl_2 _in 10× buffer (10× concentration: 500 mM 1 M KCl, 100 mM 1 M Tris HCl pH 8.4, 1% Triton-X, 15 mM MgCl_2_), each primer at 0.4 μM, 4 μl of bovine serum albumin (10 mg ml^-1^), and 0.5 μl of REDTAQ™ DNA polymerase (1 unit μl^-1^; Sigma-Aldrich Inc., St. Louis, MO). Between twenty-five and thirty cycles of PCR amplification were performed for the environmental swab samples and the bacterial colony samples. We used the lowest numbers of cycles that yielded a visible band on an agarose gel in order to minimize over-amplification of rare sequences and production of chimeric sequences. All PCR cycles included an initial denaturation step at 94°C for 1 min, an annealing step at 55°C for 45 sec and an extension step at 72°C for 1.5 min. The amplification cycles were preceded by a one-time denaturing step at 94°C for 5 min prior to the first cycle and included a final 72°C extension for 10 min to ensure complete extension for efficient cloning. Products were cleaned using Qiagen's QIAquick PCR Purification Kit.

### Cloning and Sequencing

The cleaned PCR products were cloned using the TOPO TA Cloning Kit for Sequencing (Invitrogen™, Carlsbad, CA) according to the manufacturer's instructions. Transformed One Shot chemically competent *E.coli *cells were plated on LB-agar plates containing 50 μg ml^-1 ^ampicillin and top plated with X-gal and IPTG. Next, colonies with inserts were randomly selected with a sterile toothpick and grown overnight at 37°C in 150 μl of LB broth (Fisher Biotech, Fair Lawn, NJ) containing 6% glycerol, and 1 μM ampicillin in a 96-well plate. Subsequent to cloning, a PCR amplification was performed on each of the 96 wells. The universal bacterial primers M13F (5'TTATGTAAAACGACGGCCAGT) and M13R (5'GGAAACAGCTATGACCATG) were used. Sequencing of PCR products was completed by the San Diego State MicroChemical Core Facility using an ABI 377 DNA sequencer.

### Database and Phylogenetic Analyses

The sequence chromatogram files were imported and analyzed using XplorSeq 2.0, a program written by Dr. Dan Frank at the University of Colorado (unpublished). XplorSeq imports chromatograms and determines the quality of the sequence using automatic base calling software [61]. The program also processes a batch BLAST through the NCBI database and outputs files that can easily be transferred to Microsoft Excel or the sequences exported as text files in the Fasta format.

We used the Fastgroup II program [62], to trim the 3' end of all the cleaned sequences, count replicate sequences for determining abundance of clones in libraries, and to identify a single representative for further alignment and phylogenetic analysis. The count data also allowed us to estimate the sequence coverage for each library. Coverage (C) was calculated using the following equation,

C = 1 - n/N

, where *n *is the number of unique OTU sequences observed and *N *is the total number of OTUs (i.e., sum of unique OTUs plus OTUs observed more than once) [63].

After Fastgroup analysis, the sequences were aligned using the NAST alignment software [64]. This program aligns rRNA gene sequences to a diverse set of full-length rRNA gene sequences that have been rigorously aligned using the RNA secondary structure. From here the aligned sequences were imported into the ARB application [65]. Bacterial colony species identifications were completed using a combination of BLAST results and phylogenetic analysis using the ARB program. Clone library sequences and other sequences identified in GenBank were aligned using ARB and exported as Nexus files for phylogenetic analysis using PAUP* [66] and MrBayes version 3.1.2 [67].

Phylogenetic analyses were performed with two different data sets (see Fig. 1 and Fig. 2). For each of the data sets, trees were constructed using three different methods: Bayesian, Maximum Parsimony (MP) and Maximum Likelihood (ML). The MODELTEST program [68] was used to choose the DNA substitution model that best fit our particular dataset. Bayesian analyses were performed using the General Time Reversible model [69] with a gamma-distributed among-site substitution rate heterogeneity and a fraction of sites constrained to be invariable (GTR+I+G).

All Bayesian analyses were done with four independent Markov chains run for 3,000,000 MCMC generations. Trees were sampled every 200 generations with a burn-in of 2000 trees. The best Maximum Parsimony (MP) tree, or set of trees, was found through a random addition sequence heuristic search strategy with 100 replicates. The maximum number of trees kept during each search was capped at 1000. For the MP bootstrap analyses, we performed MP searches on 100 bootstrap replicated datasets using the same heuristic search strategy except with 10, rather than 100, search replicates. We also performed a Maximum Likelihood (ML) analysis using the GTR+I+G model of evolution and a random addition sequence heuristic search strategy with 10 replicates to find the highest likelihood tree.

## Authors' contributions

LL collected half of the samples; performed most of the culturing; and identified isolates based on 16S rRNA gene sequencing. LL also created 3 of the 9 clone libraries; cleaned, edited and aligned all the sequence data; performed much of the Bioinformatics analyses and wrote early drafts of the manuscript. ST collected the other half of the samples, created 6 of the 9 clone libraries and helped with the sequence analyses. STK designed the study, wrote and edited the manuscript, completed the phylogenetic analyses and created the figures. All authors read and approved the final manuscript.
